# ChREBP Regulates Itself and Metabolic Genes Implicated in Lipid Accumulation in β–Cell Line

**DOI:** 10.1371/journal.pone.0147411

**Published:** 2016-01-25

**Authors:** Chanachai Sae-Lee, Kanya Moolsuwan, Lawrence Chan, Naravat Poungvarin

**Affiliations:** 1 Clinical Molecular Pathology Laboratory, Department of Clinical Pathology, Faculty of Medicine Siriraj Hospital, Mahidol University, Bangkok, Thailand; 2 Molecular Medicine Program, Multidisciplinary Unit, Faculty of Science, Mahidol University, Bangkok, Thailand; 3 Department of Medicine, Baylor College of Medicine, Houston, Texas, United States of America; INRA, FRANCE

## Abstract

Carbohydrate response element binding protein (ChREBP) is an important transcription factor that regulates a variety of glucose-responsive genes in hepatocytes. To date, only two natural isoforms, Chrebpα and Chrebpβ, have been identified. Although ChREBP is known to be expressed in pancreatic β cells, most of the glucose-responsive genes have never been verified as ChREBP targets in this organ. We aimed to explore the impact of ChREBP expression on regulating genes linked to accumulation of lipid droplets, a typical feature of β-cell glucotoxicity. We assessed gene expression in 832/13 cells overexpressing constitutively active ChREBP (caChREBP), truncated ChREBP with nearly identical amino acid sequence to Chrebpβ, or dominant negative ChREBP (dnChREBP). Among multiple ChREBP-controlled genes, ChREBP was sufficient and necessary for regulation of *Eno1*, *Pklr*, *Mdh1*, *Me1*, *Pdha1*, *Acly*, *Acaca*, *Fasn*, *Elovl6*, *Gpd1*, *Cpt1a*, *Rgs16*, *Mid1ip1*,*Txnip*, and *Chrebpβ*. Expression of *Chrebpα* and *Srebp1c* were not changed by caChREBP or dnChREBP. We identified functional ChREBP binding sequences that were located on the promoters of *Chrebpβ* and *Rgs16*. We also showed that *Rgs16* overexpression lead to increased considerable amounts of lipids in 832/13 cells. This phenotype was accompanied by reduction of *Cpt1a* expression and slight induction of *Fasn* and *Pklr* gene in these cells. In summary, we conclude that Chrebpβ modulates its own expression, not that of Chrebpα; it also regulates the expression of several metabolic genes in β-cells without affecting SREBP-1c dependent regulation. We also demonstrate that *Rgs16* is one of the ChREBP-controlled genes that potentiate accumulation of lipid droplets in β-cells.

## Introduction

Expression of glycolytic and lipogenic genes, including L-type pyruvate kinase (*Pklr*), acetyl-CoA carboxylase alpha (*Acaca*), thyroid hormone responsive (*Thrsp*) and fatty acid synthase (*Fasn*), is known to be regulated by glucose [1–5]. Promoters of these genes contain carbohydrate response element (ChoRE or ChRE) which is crucial for their glucose regulation [4, 6–12]. The ChoRE consists of two E-box motifs with CACGTG sequence separated by five nucleotides. In 2001, a transcription factor which recognizes ChoRE was firstly isolated from rat liver extract [13]. This basic helix-loop-helix leucine zipper transcription factor, namely carbohydrate response element binding protein (ChREBP), is highly expressed in hepatocytes, adipocytes and pancreatic β-cells [13–15]. ChREBP is activated in response to high glucose. Prolonged high glucose induces its mRNA expression, translocation from the cytosol to the nucleus, transactivation activity and DNA binding activity [13, 16–19]. Several other target genes involved in glucose metabolism and lipogenesis as well as their ChoREs have been identified, i.e. glycerol-3-phosphate dehydrogenase 1 (*Gpd1*) [11, 20], G0/G1 switch 2 (*G0S2*) [11, 20], glucose-6-phosphatase catalytic subunit (*G6pc*) [11, 21], solute carrier family 2 (facilitated glucose transporter) member 4 (*Slc2a4*) [11, 20], glucokinase (hexokinase 4) regulator (*Gckr*) [11, 20], pyruvate carboxylase (*Pc*) [11, 22, 23], glyceraldehyde 3-phosphate dehydrogenase (*Gapdh*) [11, 24], fibroblast growth factor 21 (*Fgf21*) [11, 25], and deleted in esophageal cancer 1 (*Dec1*) [26]. It has been newly discovered that glucose also regulates expression of *Chrebpβ*, naturally occurring constitutively active ChREBP variant, through a ChoRE on the exon 1b [15].

In liver cells, ChREBP involved in hepatic *de novo* lipogenesis. Overexpression of ChREBP in liver induces the expression of *Pklr*, *Fasn*, *Acaca*, stearoyl-CoA desaturase-1 (*Scd1*), and ELOVL fatty acid elongase 6 (*Elovl6*), modifying lipid composition in the process [27]. Homozygous global ChREBP knockout mice exhibited significantly decreased *de novo* fatty acid synthesis and overall adiposity [28]. In addition, overexpression of dominant negative form of ChREBP dimerization partner Mlx (Max-like protein X) downregulates *Pklr*, *Acaca*, *Fasn*, and *Elovl6* in hepatocytes and reduces intracellular triglyceride content [29].

Our previous study with pancreatic β-cells demonstrated that ChREBP deleteriously affects cell function and survival [30]. Constitutively active ChREBP (caChREBP) is a glucose-independent active mutant of ChREBP generated by deletion of the N-terminal low glucose inhibitory domain (the LID domain); its induced expression causes accumulation of neutral lipids in INS-1-derived 832/13 pancreatic β-cell line. Conversely, siRNA-mediated ChREBP silencing significantly reduces triglyceride in these cells [30]. Until now, only a few studies have explored this effect of ChREBP on accumulation of lipid droplets, an important characteristic of glucotoxicity, in pancreatic β-cells. The changes in the amount of intracellular lipid by ChREBP may be partially explained by up-regulated expression of *Fasn*, which encodes a key enzyme in *de novo* lipogenesis. ChREBP was shown to bind to both proximal and distal promoters of *Fasn* gene in β-cells [6, 31]. Microinjection of anti-ChREBP antibody in MIN6 mouse insulinoma cells blunted induction of its promoter activity by high glucose. Knockdown of ChREBP also inhibited high glucose-induced expression of *Fasn* gene. These findings have been corroborated by our previous work using 832/13 rat insulinoma cells that overexpression of caChREBP led to significant *Fasn* upregulation [30].

In this study, we aimed to further explore molecular mechanism of ChREBP-mediated lipid accumulation in pancreatic β-cells. We examined the effect of this transcription factor on expression of genes encoding enzymes of glucose metabolism and key lipogenic genes and isoforms of ChREBP itself as well.

## Materials and Methods

### Cell Culture

We cultured INS-1-derived 832/13 rat insulinoma cells (a generous gift of Dr. C. Newgard, Duke University, Durhanm, NC, USA) [32] in Roswell Park Memorial Institute (RPMI) medium (Life Technologies) supplemented with INS-1 solution, 10% fetal bovine serum (FBS) (Biochrom), 1X penicillin-streptomycin (Merck Millipore), at 37°C in a 5% CO_2_ humidified atmosphere [32].

### Plasmid construction

For the Tet-on inducible system, we replaced AgeI-MluI fragment of pTRIPZ self-inactivating (SIN) lentiviral vector (Open Biosystems) with nuclear form of enhanced yellow fluorescent protein (eYFPnuc), N-terminal Myc tagged constitutively active ChREBP (caChREBP), N-terminal Myc tagged dominant negative ChREBP (dnChREBP), or N-terminal Myc tagged regulator of G-protein signaling 16 (Rgs16). For luciferase reporter constructs, we annealed oligonucleotides containing two copies of putative ChoREs from promoters of *Chrebpβ*, *Rgs16* or *Gpd1* and ligated to pGLuc-Basic vector (New England Biolabs) with minimal TATA promoter. *Rgs16* promoter (-1519 to +159) and mutated *Rsg16* promoter with ChoRE deletion were amplified from genomic DNA extracted from 832/13 cells and cloned into pTRIPZ containing eYFPnuc. We constructed inducible lentiviral vectors that contain a single copy of optimized microRNA-adapted short hairpin RNA [33]. The target sequence for rat *Chrebpβ* was CCCAAGCCCGGCTTTTAGA. All constructs were confirmed by sequencing.

### Generation of stable tetracycline inducible cell lines

We transfect inducible lentiviral vector constructs, psPAX2 and pMD2.G vectors to human embryonic kidney 293T (HEK293T) cells using calcium phosphate method. Lentivirus were transduced into 832/13 cells in the presence of polybrene (Sigma). Infected cells were selected with puromycin (Sigma).

### RNA extraction and reverse transcription

We lysed cells after induction with 1 μg/mL doxycycline (Bio Basic). We followed manufacturer’s protocol to isolate total RNA from these lysates by RNeasy Plus Kit (Qiagen). Then, we assessed total RNA using FLUOstar Omega instrument with LVis Plate (BMG Labtech). We used Omniscript RT kit (Qiagen), random primers and 1 μg of total RNA to make cDNA in a volume of 20 μL, according to the manufacturer’s instruction.

### Quantitative PCR

We quantitated gene expression using gene-specific primers and KAPA SYBR green PCR master mix (KAPAbiosystem) on Roter-GeneQ (Qiagen) or LightCycler 480 (Roche Diagnostics). We analyzed expression of 9 housekeeping genes, i.e. eukaryotic translation elongation factor 1 gamma (*Eef1g*), hydroxymethylbilane synthase (*Hmbs*), delta-aminolevulinate synthase 1 (*Alas1*), ribosomal protein L13a (*Rpl13a*), tyrosine 3-monooxygenase/tryptophan 5-monooxygenase activation protein, zeta (*Ywhaz*), hypoxanthine phosphoribosyltransferase 1 (*Hprt1*), beta-actin (*Actb*), 18s ribosomal RNA (*Rns18*), and peptidylprolyl isomerase A (cyclophilin A) (*Ppia*). We selected three most stable housekeeping genes of each experimental setting for normalization of gene expression based on geNorm analysis [34]. Primer sequences used in this study are available upon request.

### Sequence alignment

We aligned the amino acid sequence in MegAlign module of DNASTAR software (Lasergene). We identified multi-species conserved regulatory sequences by GenomeVISTA (http://genome.lbl.gov/vista/) for ChREBPβ and Gpd1, and UCSC Genome Browser (https://genome.ucsc.edu/) for Rgs16.

### Luciferase assay

We transiently transfected three plasmid DNA constructs, *Gaussia* luciferase construct containing putative ChREBP binding elements, cytomegalovirus promoter-driven *Cypridina* luciferase construct, and caChREBP construct or empty vector, to 832/13 cells in 96-well plates by Lipofectamine2000 (Life Technologies). We collected culture media at 48 hours after transfection. We then sequentially detected secreted luciferases using *Gaussia* luciferase and *Cypridina* luciferase assay kits (New England Biolabs). Luciferase expressed from *Gaussia* luciferase reporter plasmid is normalized by the activity of *Cypridina* luciferase.

### Oil Red O Staining

We washed with phosphate-buffered saline after induction by 1 μg/mL doxycycline, and fixed these cells in 10% neutral buffered formaldehyde for 2 hour. We then removed formaldehyde and washed the cells with water. We added 0.3% Oil Red O in isopropanol to the fixed cells. We removed Oil Red O solution and washed the cells with water. Stained neutral lipid droplets were visualized and photographed under the IX81 inverted microscope (Olympus). Areas of the lipid droplets were quantified using Adobe Photoshop 9.0 CS2.

### Statistical analysis

We presented all data as the mean ± standard deviation (SD) between three biological replicates (expression) or standard error of the mean (SEM) of four determinations from two independent experiments (luciferase assay). We compared sample groups by Student’s two-tailed *t*-test. The *p*<0.05 was considered statistically significant.

## Result

### *Chrebpβ* stimulates its own expression via a feed-forward loop

**Fig 1:**
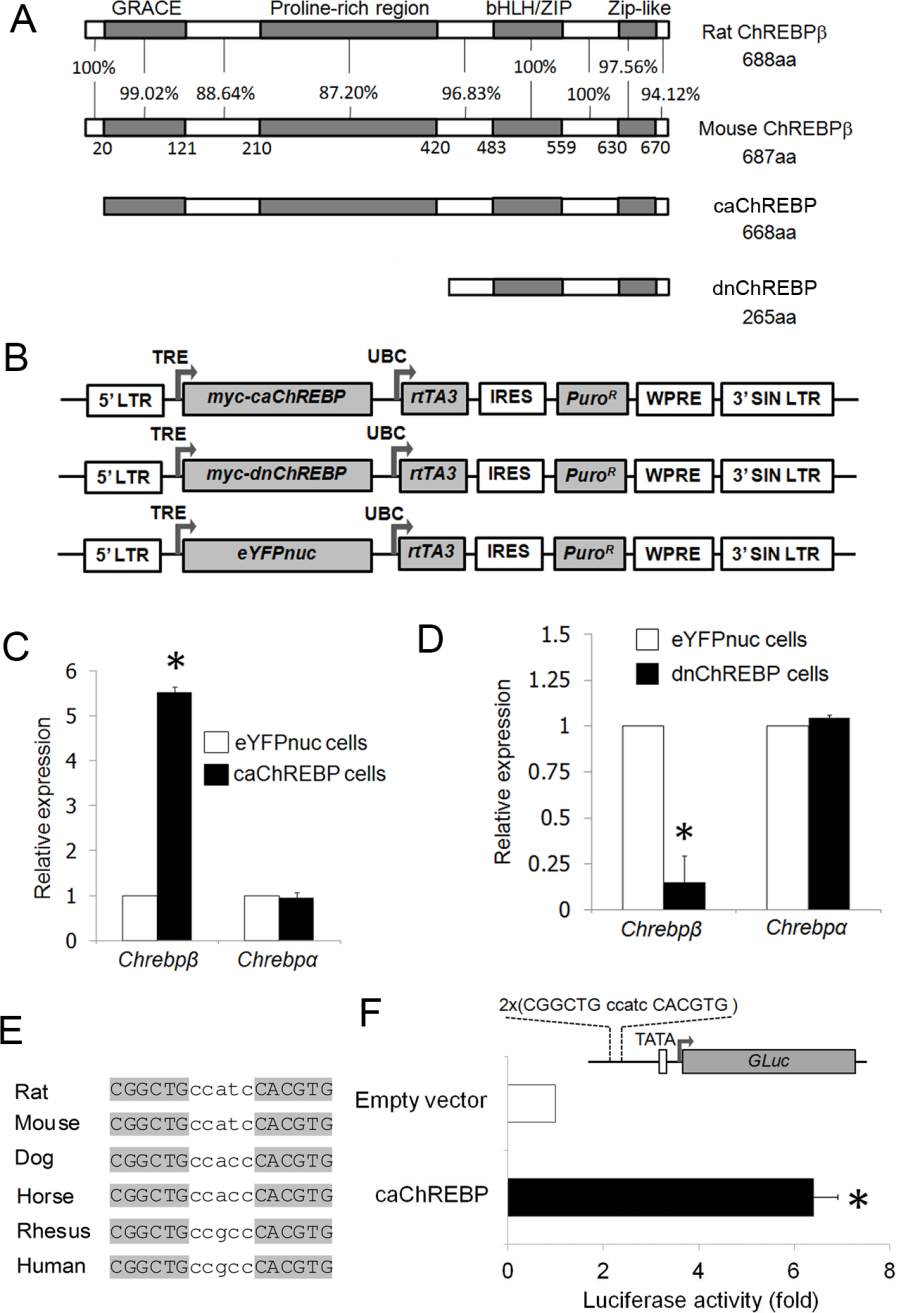
Exogenous ChREBP regulates *Chrebpβ* expression. (A) Amino acid alignment among rat ChREBPβ, mouse ChREBPβ, caChREBP, and dnChREBP. GRACE, glucose response activation conserved element; bHLH, basic Helix-Loop-Helix-Leucine. (B) Schematic diagram of tetracycline-inducible vectors for overexpression of *caChREBP*, *dnChREBP*, and *eYFPnuc*. LTR, long terminal repeat; TRE, tetracycline-responsive promoter element; UBC, human ubiquitin C promoter; *rtTA3*, reverse tetracycline-transactivator 3; IRES, internal ribosomal entry site; *Puro*^*R*^, puromycin resistance gene; WPRE, Woodchuck hepatitis posttranscriptional regulatory element; SIN LTR, self-inactivating long terminal repeat. (C) caChREBP induces *Chrebpβ* expression. We incubated caChREBP cells for 48h in RPMI with 11 mmol/l D-glucose in the presence of doxycycline 1 μg/mL. We isolated the RNA and performed RT-qPCR using ChREBP isoform-specific primers. The histograms are the means of relative RNA levels normalized to *Eef1g* and *Rpl13a* and expressed as fold activation over the activity seen in eYFPnuc cells incubated in RPMI with 11 mmol/l D-glucose in the presence of doxycycline 1 μg/mL. *, p< 0.05 compared with eYFPnuc cells. (D) dnChREBP reduces the *Chrebpβ* expression. We preincubated dnChREBP cells for 24h in RPMI with 5.5 mmol/l D-glucose in the presence of doxycycline 1 μg/mL to minimize expression of endogenous nuclear ChREBP and let the induced dnChREBP occupy ChoREs and switched to in RPMI with 25 mmol/l D-glucose in the presence of doxycycline 1 μg/mL for 48h to induce strong expression and activity of endogenous nuclear ChREBP. We isolated the RNA and performed RT-qPCR using ChREBP isoform-specific primers. The histograms are the means of relative RNA levels normalized to *Rns18* and *Hprt1* and expressed as fold activation over the activity seen in eYFPnuc cells incubated under the same condition. *, p< 0.05 compared with eYFPnuc cells. (E) Alignment of ChoRE sequence presents in *Chrebpβ* promoter among rat (at the position -109 to -93), mouse, dog, horse, rhesus, and human. Sequence assemblies and coordinates are as follows: Rat: Mar.2012 Chr12(-): 26638089–26638073, Mouse: Jul.2007 Chr5(+): 135565651–135565667, Rhesus: Jan.2006 Chr3(-):51178915–51178899, Horse: Jan.2007 ChrUn(-):175282430–175282414, Dog: May 2005 Chr6(+): 9619423–9619439, Human: Mar.2006 Chr7(-):72700300–72700284. Color coding: light grey, identical residues; dark grey, unconserved residues. (F) Functional analysis of putative ChoRE sequences at the position -109 to -92 on *Chrebpβ*. We co-transfected luciferase reporter driven by two copies of rat *Chrebpβ* ChoRE upstream of minimal TATA promoter with caChREBP in 832/13 cells in RPMI with 5.5 mmol/l D-glucose. *Gaussia* luciferase activity was measured at 48h and normalized to *Cypridina* luciferase, and expressed as fold activation over the activity seen in cells transfected with two copies of ChoRE with minimal TATA promoter and empty vector. *, p< 0.05.

We previously demonstrated that constitutively active ChREBP (caChREBP), mouse ChREBP with deletion of low glucose inhibitory domain, induces apparent β-cell apoptosis by upregulation of thioredoxin-interacting protein (*Txnip*) [30]. This caChREBP is highly similar to mouse ChREBPβ, which is 19 amino acids longer at the N-terminal end (Fig 1A), both being characterized by the absence of the LID domain conferring them constitutive activity. To overcome a problem with cell lost in stable transgene overexpressing cells, we adopted Tet-on system with reverse tetracycline transactivator 3 (rtTA3), which offers minimal leakiness and high inducibility [35]. We established inducible 832/13 pancreatic β-cell lines for caChREBP (caChREBP cells) and dominant negative ChREBP lacking transactivation domain (dnChREBP cells) using self-inactivating lentiviral vector (Fig 1B). Since both caChREBP and dnChREBP are located in the nucleus, nuclear form of enhanced yellow fluorescent protein (eYFPnuc cells) was chosen to establish control cells.

We performed reverse transcription followed by the quantitative polymerase chain reaction (RT-qPCR) and found that expression of all ChREBP mutants were successfully induced by addition of doxycycline in culture media (data not shown). High levels of caChREBP and dnChREBP expression were reached after 48–72 hours of induction. We found that endogenous rat *Chrebpβ* expression was significantly increased in caChREBP cells by ~5.5–fold as compared to expression in eYFPnuc cells, while expression of endogenous *Chrebpα* did not change (Fig 1C). In contrast, *Chrebpβ* expression was decreased significantly by ~85% in dnChREBP cells, while *Chrebpα* expression also failed to show a change in this setting (Fig 1D).

Herman et al identified a ChoRE at the position +158 to +174 on exon1b of mouse *Chrebp* that mediates specific induction of *Chrebpβ* expression by *Chrebpα* [15]. This sequence is partially conserved from rat to human. In addition, a separate E-box at the position -98 to -93 also conferred glucose responsiveness of proximal mouse *Chrebpβ* promoter. We noted that deletion of this E-box obviously abolished high glucose-induced promoter activity in the presence of *Chrebpα* and *Mlx* in their luciferase assay data, whereas deletion of ChoRE had minor effect on enhancing promoter activity by glucose, a fact that is consistent with a crucial role of this E-box in regulation of *Chrebpβ* transcription. We observed the presence of another E-box-liked sequence separated from the first E-box by 5 base pairs (Fig 1E). The sequence of this newly identified putative ChoRE is highly conserved from rat to human, supporting a functionally important role for *Chrebpβ* expression. When this manuscript was under review, Zhang et al concomitantly found the same ChoRE sequence and demonstrated that ChREBP binds to this ChoRE [36]. This element is necessary for *Chrebpβ* promoter in response to glucose in 832/13 cells.

To further explore whether both ChoREs are sufficient for the activation by ChREBP and are equally preferred to caChREBP, we generated two luciferase reporter constructs driven by two copies of rat ChoRE located upstream of minimal TATA promoter and performed luciferase assay. We found that co-transfection of caChREBP construct and luciferase reporter construct with two copies of the rat sequence at the position +160 to +176 of *Chrebpβ*, that is conserved to previously identified mouse *Chrebpβ* ChoRE, did not change luciferase reporter compared with empty vector in 832/13 cells (data not shown). Interestingly, caChREBP gave 6.4-fold stimulation of activity of promoter containing two copies of rat *Chrebpβ* ChoRE sequence identified in this study (position -109 to -92) (Fig 1F). Given the fact that amino acid sequences of our caChREBP is almost identical to endogenous *Chrebpβ* in mouse and rat (Fig 1A) and caChREBP stimulates expression of endogenous *Chrebpβ*, our finding provides a clue to an autoregulation of *Chrebpβ* via recently recognized ChoRE sequence on its proximal promoter.

### ChREBP regulates metabolic enzyme genes and lipogenic genes in β-cells independent of SREBP-1c

**Fig 2:**
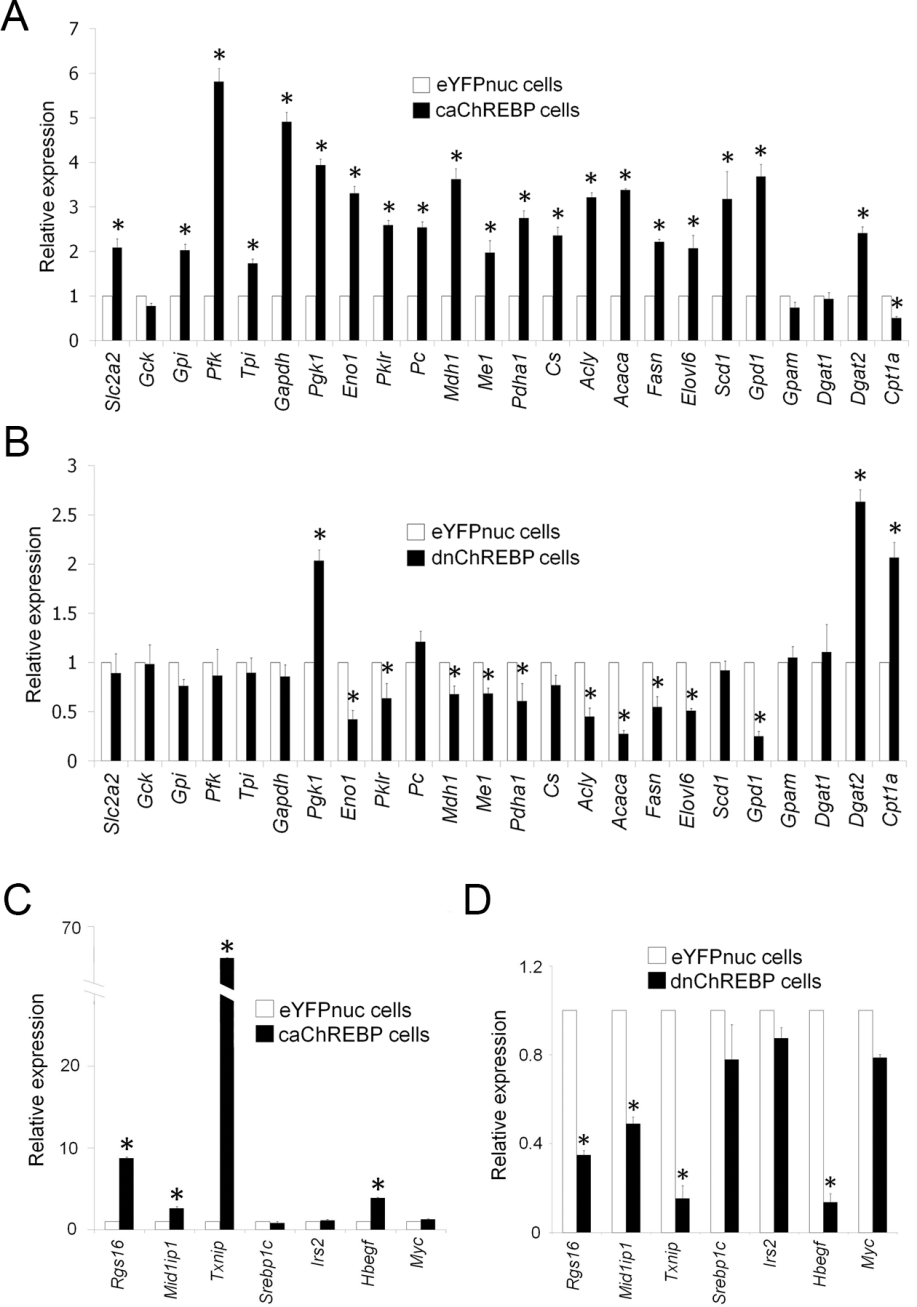
ChREBP regulates the expression of metabolic genes in 832/13 β-cells. (A-B) Expression of genes encoding enzymes involved in glycolysis, lipogenesis, triglyceride biosynthesis and lipid oxidation in caChREBP cells (A) and dnChREBP cells (B). *p< 0.05 compared with eYFPnuc cells. (C-D) Expression of metabolic genes, including *Rgs16* and *Mid1ip1*, *Srebp1c* and its target *Irs2*, and genes involved in cell proliferation, *Hbegf* and *Myc*, in caChREBP cells (C) and dnChREBP cells (D). *p< 0.05 compared with eYFPnuc cells.

We, and Da Silva Xavier et al, have shown that ChREBP controls *Pklr* and *Fasn* expression and intracellular triglyceride levels in rat and mouse pancreatic β-cell lines [30, 31]. In order to get a more comprehensive picture on ChREBP-regulated genes in β-cells, we first performed RT-qPCR analysis of expression of metabolic pathway gene panels involved in glycolysis, lipogenesis, and lipid oxidation in control and caChREBP 832/13 cells (Fig 2A). We found that not only were the expression levels of *Pklr* and *Fasn* upregulated in caChREBP cells, but expression of many glycolytic and lipogenic genes were also significantly increased, i.e. solute carrier family 2 (facilitated glucose transporter), member 2 (*Slc2a2*), glucose-6-phosphate isomerase (*Gpi*), phosphofructokinase (*Pfk*), Triosephosphate Isomerase 1 (*Tpi1*), *Gapdh*, phosphoglycerate kinase1 (*Pgk1*), enolase1 (*Eno1*), *Pc*, malate dehydrogenase 1 (*Mdh1*), malic enzyme1 (*Me1*), pyruvate dehydrogenase (lipoamide) alpha 1 (*Pdha1*), citrate synthase (*Cs*), ATP citrate lyase (*Acly*), *Acaca*, *Elovl6*, *Scd1*, *Gpd1*, and diacylglycerol o-acyltransferase 2 (*Dgat2*). Carnitine palmitoyltransferase 1 (*Cpt1a*), a gene associated with lipid oxidation, was reduced significantly.

We next examined the expression level of these genes in dnChREBP cells. Our dnChREBP, a mouse ChREBP mutant lacking of transactivation domain, occupies ChoRE without transcriptional induction of target genes (data not shown). We found that levels of gene expression of *Eno1*, *Pklr*, *Mdh1*, *Me1*, *Pdha1*, *Acly*, *Acaca*, *Fasn*, *Elovl6*, and *Gpd1*were downregulated after induction of dnChREBP expression for 36–72 hours (Fig 2B). Expression of *Pgk1*, *Dgat2* and *Cpt1a* was increased significantly.

We also evaluated the expression of other lipogenic genes, including *Rgs16*, MID1 interacting protein 1 *(Mid1ip1)*, and *Txnip* [37–39], and found that these genes were induced in caChREBP cells (Fig 2C) and were reduced in dnChREBP cells (Fig 2D). Sterol regulatory element-binding protein-1c (SREBP-1c) is a key transcription factor that shares a part of downstream target genes with ChREBP, e.g. *Pklr*, *Fasn* and *Acaca*, and controls hepatic fatty acid synthesis [40]. It has been reported that ChREBP binds to human SREBP-1c gene and conveys glucose action on SREBP-1c gene expression in hepatocytes [41]. In INS-1 β-cell line, overexpression of nuclear active form of SREBP-1c led to marked accumulation of lipid droplets [42]. To exclude the possibility that ChREBP indirectly affected expression of lipogenic genes via SREBP-1c, we examined the expression of *Srebp1c* using variant-specific primers and found that *Srebp1c* gene expression was not altered in caChREBP or dnChREBP cells (Fig 2C and 2D). In addition, expression of insulin receptor substrate 2 (*Irs2*), a *Srebp1c* target in β-cells [43], did not change in either type of cells. These findings suggest that ChREBP controls downstream glycolytic and lipogenic genes independent of SREBP-1c.

Recently published article reports that ChREBP controls expression of heparin-binding EGF-like growth factor (*Hbegf*) and *Myc* genes which are involved in cellular proliferation [36]. In our study, we found that *Hbegf* expression is significantly increased in caChREBP cells and decreased in dnChREBP cells (Fig 2C and 2D), whereas the expression of *Myc* remained unchanged in the two types of cells.

### Evolutionarily conserved *Gpd1* ChoRE sequence does not respond to ChREBP in β-cells

*Gpd1* is another gene whose expression was increased by caChREBP and decreased by dnChREBP in this study. In hepatocytes, *Gpd1* was induced by glucose and this induction was repressed by dnMlx [20]. A potential ChoRE on was identified at position -1943 to -1927 of the rat *Gpd1* promoter and binding of ChREBP to this ChoRE was confirmed by electrophoretic mobility shift assay [20]. However, glucose cannot activate either ChoRE-containing minimal *Gpd1* promoter or minimal TATA-containing promoter with two copies of *Gpd1* ChoRE [20].

**Fig 3:**
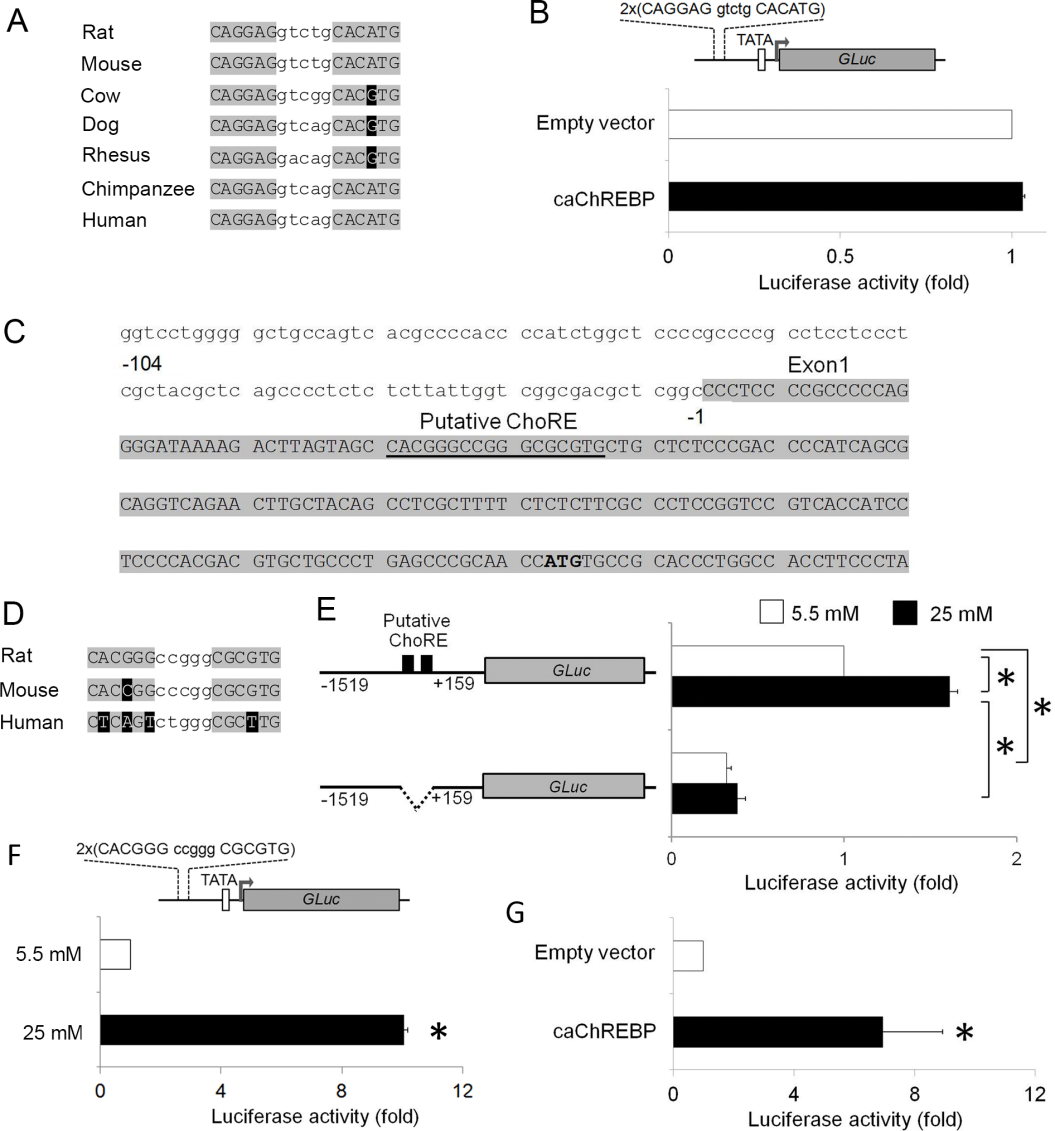
Effect of caChREBP on *Gpd1* or *Rgs16* ChoRE-containing promoters. (A) Alignment of ChoRE sequence presented on *Gpd1* promoters among rat (at the position -1943 to -1927), mouse, cow, dog, rhesus, chimpanzee, and human. Sequence assemblies and coordinates are as follows: Rat: Mar.2012 ChrX(+): 115873245–11587361, Mouse: Dec.2011 Chr15(+): 99716109–99716125, Cow: Oct.2011 Chr5(-): 32732081–32732065, Dog: Sep.2011 Chr27(-): 4623314–4623298, Rhesus: Oct.2010 Chr11(+): 47359512–47359528, Chimpanzee: Feb.2011 Chr12(-): 39238594–39238578, and Human: Feb.2009 Chr12(+): 50496575–50496591. Color coding: light grey, identical residues; dark grey, unconserved residues. (B) caChREBP cannot activate *Gpd1* ChoRE-containing promoter. We co-transfected luciferase reporter driven by two copies of rat *Gpd1* ChoRE (position -1943 to -1927 relative to TSS) upstream of minimal TATA promoter with caChREBP in 832/13 cells in RPMI with 5.5 mmol/l D-glucose. Gaussia luciferase activity was measured at 48h and normalized to Cypridina luciferase, and expressed as fold activation over the activity seen in cells transfected with two copies of ChoRE with minimal TATA promoter and empty vector. *, p< 0.05. (C) Sequence of rat Rgs16 proximal promoter (-104 to +196, relative to TSS). Bold text, Start codon. Color coding: light grey, Exon 1. (D) Alignment of ChoRE sequence presented on *Rgs16* promoter among rat (at the position +37 to +53), mouse, and human. Sequence assemblies and coordinates are as follows: Rat: Mar.2012 Chr13(+): 76145198–76145214, Mouse: Dec.2011 Chr1(+): 153740341–153740357, and Human: Dec.2013 Chr1(-): 182604401–182604385. Color coding: light grey, identical residues; dark grey, unconserved residues. (E) The effect of high glucose on the activity of natural *Rgs16* promoter and mutated *Rgs16* promoter. We transfected luciferase reporter driven by natural *Rgs16* promoter (position -1519 to +159 relative to TSS) or ChoRE-deleted *Rgs16* promoter in 832/13 cells cultured in RPMI with 5.5 mmol/l or 25 mmol/l D-glucose. Gaussia luciferase activity was measured at 48h and normalized to Cypridina luciferase, and expressed as fold activation over the activity seen in cells transfected with natural *Rgs16* promoter and exposed to 5.5 mmol/l D-glucose. *, p< 0.05. (F-G) Glucose and caChREBP stimulates *Rgs16* ChoRE-containing promoter. We transfected luciferase reporter driven by two copies of rat *Rgs16* ChoRE (position +37 to +53 relative to TSS) upstream of minimal TATA promoter in 832/13 cells in RPMI with 5.5 or 25 mmol/l D-glucose (F) or co-transfected with caChREBP or empty vector in 832/13 cells in RPMI with 5.5 mmol/l D-glucose (G). Gaussia luciferase activity was measured at 48h and normalized to Cypridina luciferase, and expressed as fold activation over the activity seen in cells transfected with two copies of ChoRE with minimal TATA promoter and exposed to 5.5 mmol/l D-glucose (F) or co-transfected with empty vector (G). *, p< 0.05.

We found that *Gpd1* ChoRE is highly conserved not only between rodents and human, but also in several other species (Fig 3A). To evaluate whether this *Gpd1* ChoRE can respond to ChREBP in β-cells, we cloned two copies of *Gpd1* ChoRE upstream of the minimal TATA promoter in the reporter plasmid and performed luciferase assay in the existence of caChREBP or empty vector in 832/13 cells. We found that caChREBP had no impact at all on activity of this promoter (Fig 3B). Our finding supports a previous report that ChREBP does not regulate *Gpd1* expression through this putative ChoRE [20].

### Proximal promoter of *Rgs16* contains functional ChoRE sequence

Among the genes that are altered in both caChREBP and dnChREBP cells in the current study, *Txnip* is the most affected gene by ChREBP. Functional ChREBP binding sites on human and murine Txnip promoters were well described [30, 44]. *Rgs16* is the second most upregulated gene by caChREBP (~9–fold increased). Few studies in hepatocytes have shown that ChREBP regulates *Rgs16* expression [20, 39]. Iizuka et al have demonstrated that either high glucose condition or dominant active form of ChREBP is able to induce *Rgs16* mRNA expression in 832/13 cells [45]. We have also confirmed that glucotoxic condition upregulates *Rgs16* expression in these cells. Our RT-qPCR result showed that *Rgs16* expression in 832/13 cells under 25 mmol/l D-glucose condition is ~8.9-fold higher than that in cells exposed to 5.5 mmol/l D-glucose (data not shown).

To further investigate whether ChREBPβ mediates glucose-induced *Rgs16* expression, we have constructed lentiviral vectors with Tet-inducibility to generate microRNA-adapted shRNA based on optimized miRNA mimic design (S1A Fig.) [33]. We transduced viral vectors in 832/13 cells to establish inducible cell lines harboring *Chrebpβ* shRNA targeting sequence (*mirGE-β* cells) and non-silencing sequence (*mirGE-N* cells). The expression of *Chrebpβ* in induced *mirGE-β* cells was ~46% as compared to expression in induced *mirGE-N* cells (S1B Fig.). However, *Rgs16* expression was not different between these two cell lines.

The ChoRE on *Rgs16* promoter has never been identified or tested. We analyzed rat *Rgs16* promoter *in silico* and found a putative ChoRE sequence at position +37 to +53 from the transcription start site based on the Ensembl database (ENSRNOT00000032236) (Fig 3C). Sequence alignment of each ChoRE exhibited high conservation between rat and mouse, but not well-conserved in the human sequence (Fig 3D).

To dissect the role of a putative ChoRE in the glucose activation of the *Rgs16* promoter, we used luciferase reporter constructs driven by the *Rgs16* promoter (-1519 to +159) with or without this ChoRE (Fig 3E) to transfect into 832/13 cells under low (5.5 mmol/l) or high (25 mmol/l) glucose condition and performed luciferase assay. We found a small but significant increase (~1.6-fold) in the activity of natural *Rgs16* promoter by glucose, whereas *Rgs16* promoter without ChoRE exhibited a lower basal activity and did not respond to glucose (Fig 3E).

To test whether ChoRE sequence on the *Rgs16* promoter is able to mediate glucose- and ChREBP-induced promoter activity, we performed luciferase assay in 832/13 cells using reporter construct driven by minimal TATA promoter and 2 copies of putative rat *Rgs16* ChoRE in low glucose (5 mmol/l D-glucose) or high glucose (25 mmol/l D-glucose) condition (Fig 3F) and in the presence of caChREBP or empty vector construct (Fig 3G). We found that glucose and caChREBP induced activity of the promoter that contains *Rgs16* ChoRE at position +37 to +53 by ~10-fold and 6.9–fold, respectively (Fig 3F and 3G). Our result indicates that this sequence is both glucose- and ChREBP-responsive in pancreatic β-cells.

### Increased *Rgs16* enhances accumulation of neutral lipid in β-cells

**Fig 4:**
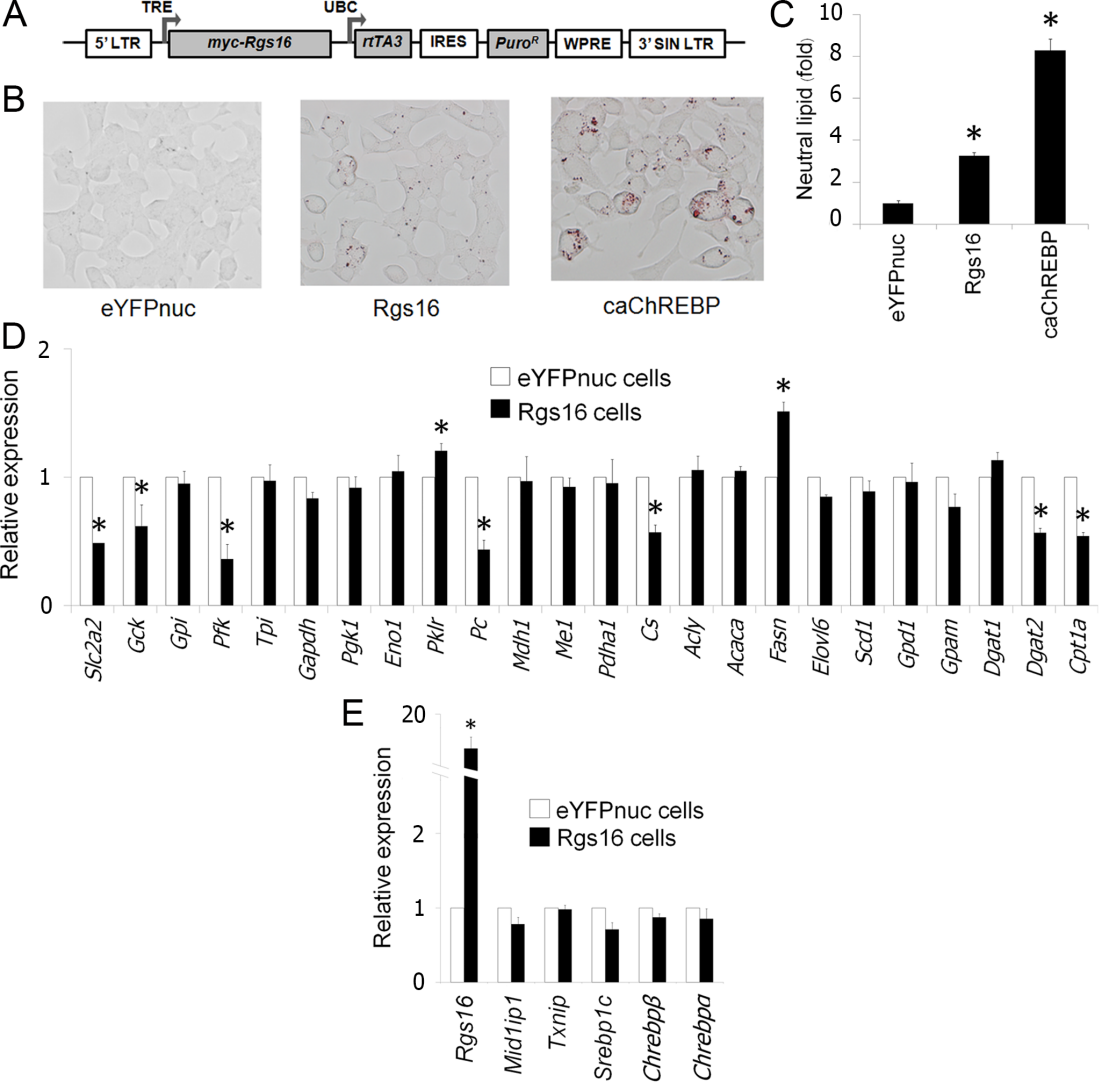
*Rgs16* enhances accumulation of neutral lipid in 832/13 β-cells. (A) Schematic diagram of tetracycline-inducible vectors for overexpression of *Rgs16*. LTR, long terminal repeat; TRE, tetracycline-responsive promoter element; UBC, human ubiquitin C promoter; *rtTA3*, reverse tetracycline-transactivator 3; IRES, internal ribosomal entry site; *Puro*^*R*^, puromycin resistance gene; WPRE, Woodchuck hepatitis posttranscriptional regulatory element; SIN LTR, self-inactivating long terminal repeat. (B-C) Overexpression of *Rgs16* triggers lipid accumulation in 832/13 cells. We incubated eYFPnuc cells, Rgs16 cells, and caChREBP cells for 72h in RPMI with 11 mmol/l D-glucose in the presence of doxycycline 1 μg/mL. We stained these cells for neutral lipid by Oil red O (B). Histograms (C) represent the amount of stained intracellular lipid compared with eYFPnuc cells. *, p< 0.05 compared with eYFPnuc cells. (D-E) Effects of *Rgs16* overexpression on genes encoding metabolic enzymes (D) and related metabolic genes (E). We incubated Rgs16 cells for 72h in RPMI with 11 mmol/l D-glucose in the presence of doxycycline 1 μg/mL. We isolated the RNA and performed RT-qPCR using gene-specific primers. The histograms are the means of relative RNA levels normalized to *Eef1g* and *Hprt1* and expressed as fold activation over the activity seen in eYFPnuc cells preincubated with 11 mmol/l D-glucose in the presence of doxycycline 1 μg/mL. *, p< 0.05 compared with eYFPnuc cells.

Transgenic mice overexpressing liver-specific Rgs16 protein had reduced hepatic fatty acid oxidation and developed hepatosteatosis [39]. To investigate whether *Rgs16* plays a similar role in β-cells, we overexpressed rat *Rgs16* using Tet-on inducible system in 832/13 cells (Fig 4A). Exogenous *Rgs16* was successfully induced after addition of doxycycline to culture medium for 72 hour (data not shown). We then performed Oil Red O staining of *Rgs16*-overexpressing cells (Rgs16 cells), caChREBP cells and eYFPnuc cells to visualize intracellular neutral lipids (Fig 4B). Quantitative analysis showed that Rgs16 cells exhibited ~3.3–fold increased cytoplasmic lipids as compared to eYFPnuc cells (Fig 4C). As we expected, caChREBP cells displayed even higher intracellular lipids, ~8.3–fold compared with eYFPnuc cells.

We next measured gene expression in Rgs16 cells by RT-qPCR (Fig 4D and 4E). Total *Rgs16* transcripts were increased ~17.0–fold (Fig 4E). Concomitantly, *Pklr* and *Fasn* were slightly upregulated, while *Cpt1a* expression was reduced to half (Fig 4D). Expression of *Slc2a2*, *Gck*, *Pfk*, *Pc*, *Cs*, and *Dgat2*, was significantly downregulated. *Chrebpα* and *Chrebpβ* expression did not change significantly in Rgs16 cells (Fig 4E).

## Discussion

ChREBP is a glucose-responsive transcription factor that was shown to play an important role in β-cell glucotoxicity [30]. Although we have shown that accumulation of lipid droplets, a major phenotypic characteristic in glucotoxic β-cells, appears to be mediated by ChREBP expression, the mechanisms underlying this phenomenon are only partly understood. Alteration in expression of metabolic genes may partially explain ChREBP-induced lipid accumulation. A few metabolic genes have been well-characterized as direct downstream targets of ChREBP in pancreatic β-cells, including *Txnip*, *Fasn*, and *Pklr*. ChREBP binds to their respective promoters, regulates promoter activities as well as expression of these genes [30, 31, 44, 45]. *Gpd1* and *Rgs16* expression was shown to be activated by a dominant active mutant of ChREBP in INS-1E β-cell line [45]. It is therefore not surprising to see upregulation of these genes by caChREBP and downregulation by dnChREBP in our experiments. Several other metabolic genes exhibited the same expression pattern, i.e. *Eno1*, *Mdh1*, *Me1*, *Pdha1*, *Acly*, *Acaca*, *Elovl6*, *Mid1ip1* and *Chrebpβ*, while a gene encoding the lipid oxidation enzyme, *Cpt1a*, manifested the opposite expression pattern. Our results suggest that ChREBP is sufficient and necessary for regulation of these genes. Among this list, *Acaca*, *Mid1ip1* and *Chrebpβ* genes are ChREBP targets with ChoRE on their promoters [4, 15, 46]. Although *Gapdh* and *Pc* are also ChREBP target genes which ChoREs have been identified [22–24], their expressions were controlled by caChREBP, but not dnChREBP, in our experiments. A similar pattern was found in expression of *Slc2a2*, *Gpi*, *Pfk*, *Cs*, *Tpi*, and *Scd1*. These findings indicate that ChREBP is sufficient but not necessary for regulation of these genes in β-cell line. *Pgk1* and *Dgat2* were upregulated by both caChREBP and dnChREBP. There may be other factors that modulate the effect of ChREBP on these genes. Despite the fact that *Srebp1c* is a candidate ChREBP target gene [41], we clearly showed that ChREBP has no influence on the expression of *Srebp1c* and its target gene *Irs2* in 832/13 β-cells, suggesting that ChREBP regulates expression of several metabolic genes independent of SREBP-1c.

Previous studies have used different approaches to demonstrate functional ChoREs in β-cells. *Txnip* and *Pklr* ChoREs confer ChREBP activation of the respective promoters in β-cells [45]. *G6pc* and *Fasn* ChoREs have been functionally analyzed in term of responsiveness to high glucose, but not ChREBP [21, 31]. In the current study, we describe two new functional ChoRE sequences on the promoters of *Chrebpβ* and *Rgs16*. In agreement with a recent study by Zhang et al. [36] that was published while our manuscript was under review, we found that the same *Chrebpβ* ChoRE sequence played a major role on the *Chrebpβ* promoter in response to glucose. The *Chrebpβ* ChoRE sequence is highly conserved. Our results further demonstrate that the caChREBP, which has similar amino acid sequence domains as *Chrebpβ*, was able to activate artificial promoters which contain 2 copies of these ChoREs. Our experiments suggest the possibility that *Chrebpβ* auto-regulates its own expression, but not *Chrebpα* expression, perhaps through ChoRE at the position -109 to -93 of rat *Chrebpβ*, that is absent in *Chrebpα*. It is noteworthy that nucleotide at the third position of this rat *Chrebpβ* sequence is guanine (G), which is inconsistent with a previously articulated hypothesis on functional ChoRE sequence used to explain the absence of *Gpd1* ChoRE responsiveness to high glucose [20]. On the other hand, our result is supported by previous reports on ChoREs of Krüppel-like factor-10 (*Klf10*) and *Fgf21* which can be activated by ChREBP and high glucose [25, 47]. It is likely that the sequence context surrounding the third nucleotide on functional ChoRE contributes to the response.

It has been shown that previously identified *Chrebpβ* ChoRE, at the position +158 to +174, mediates the activation of mouse *Chrebpβ* promoter by *Chrebpα* [15]. In our study, activity of the promoter with two copies of its conserved ChoRE sequence at position +160 to +176 of rat *Chrebpβ* did not alter. These findings cannot be directly compared to each other because there are at least four important differences in experimental design/condition: 1) our study focused on function of ChoRE independent of other response elements on *Chrebpβ* promoter. 2) unconserved fourth nucleotide of these ChoREs (C versus T nucleotide), 3) distinction between response to activation by caChREBP and Chrebpα/Mlx with glucose, and 4) difference in cell types between experiments (832/13 β-cells versus HEK293T cells).

While this manuscript was under review, Zhang et al. reported that ChREBP differentially binds to *Chrebpβ* ChoREs in a tissue-specific manner [36]. It is noteworthy that our recently published high through-put DNA sequencing (ChIP-seq) data [11] also showed that ChREBP binds to the *Chrebpβ* promoter in both mouse liver and white adipose tissue with a higher preference for the liver. Interestingly, the ChoRE identified in the current study is located at the summit of the ChIP-seq peaks in both tissues (S2 Fig); there is no evidence of ChREBP binding occurs at the position that had previously been identified as ChoRE [15]. Additional studies will be needed to further dissect the tissue specificity of ChREBP binding on two *Chrebpβ* ChoREs.

Rgs16 is one of the proteins that regulate G protein-coupled receptor signaling. It has been reported that expression of *Rgs16* is quiescent in adult pancreatic islets and chronic hyperglycemia reactivates *Rgs16* re-expression [48]. In this study, we have tried to knockdown *Chrebpβ* to determine if *Chrebpβ* mediates high glucose-induced Rgs16 expression in β-cells. We found that ~54% *Chrebpβ* silencing has no effect on *Rgs16* expression. This diminished *Chrebpβ* expression level may not be sufficient to affect target gene expression and the existing *Chrebpα* protein could activate its target genes in high glucose condition. A similar phenomenon was recently reported that ~50% *Chrebpβ* silencing could not alter *Txnip* expression in 832/13 cells [36]. Therefore it is still unclear whether *Chrebpβ* plays a major role on *Rgs16* expression in β-cells under high glucose condition.

Our study demonstrates that functional ChoRE sequence of rat *Rgs16* is located at the position +37 to +53 relative to its transcription start site. Induced expression of caChREBP increased expression of *Rgs16* in β-cells, while dnChREBP had an opposite effect. Overexpression of rat *Rgs16* led to an increase in lipid accumulation in 832/13 cells in a way analogous to that found in liver-specific *Rgs16* overexpression in mice [39]. Gene expression analysis reveals effects of *Rgs16* on expression of three genes encoding key enzymes that may mediate the accumulation of lipid droplets. Decreased expression of *Cpt1a* is similar to that found in the liver of *Rgs16* transgenic mice. It was proposed that *Rgs16* does not promote genes involved in glycolysis or fatty acid synthesis, however, expression of *Pklr* and *Fasn* was mildly increased in our *Rgs16*-overexpressing 832/13 cells. Interestingly, we observed reduced expression of *Slc2a2*, *Pfk*, *Pc*, *Cs* and *Dgat2*, opposites to what we have seen in caChREBP cells. These effects of increased *Rgs16* expression may somehow interfere with impact of caChREBP on expression of these genes in caChREBP-overexpressing 832/13 cells. Neither *Chrebpα* nor *Chrebpβ* expression was influenced by induction of *Rgs16*, indicating that no feedback loop existed.

**Fig 5:**
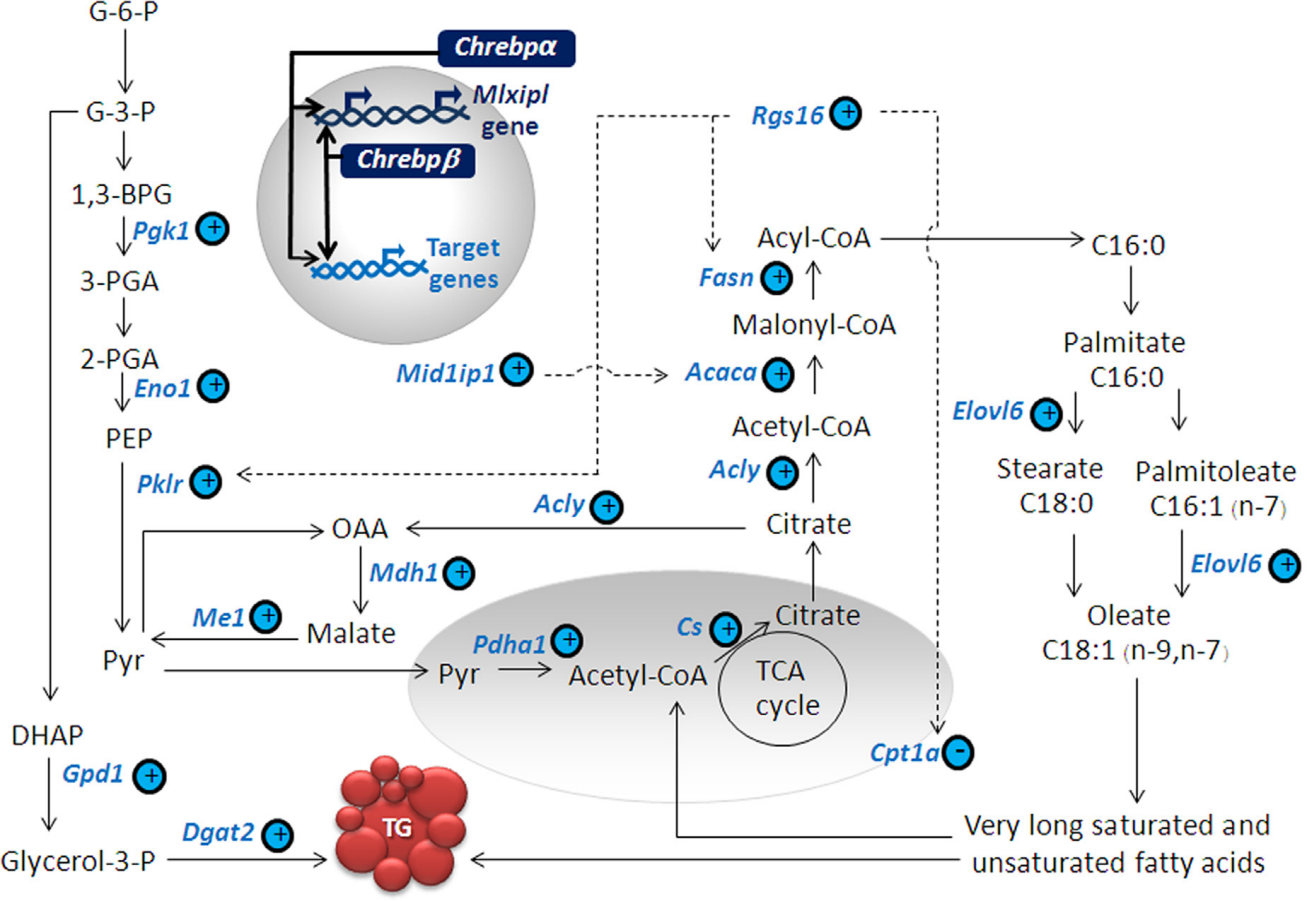
Model of ChREBP-induced lipid accumulation in β-cells. Plus symbol, upregulation; Minus symbol, downregulation; *Pgk1*, phosphoglycerate kinase1; *Eno1*, enolase1; *Pklr*, L-type pyruvate kinase; *Mdh1*, malate dehydrogenase 1; *Me1*, malic enzyme1; *Pdha1*, pyruvate dehydrogenase (lipoamide) alpha 1; *Cs*, citrate synthase; *Acly*, ATP citrate lyase; *Mid1ip1*, MID1 interacting protein 1; *Acaca*, acetyl-CoA carboxylase alpha; *Fasn*, fatty acid synthase; *Elovl6*, ELOVL fatty acid elongase 6; *Rgs16*, regulator of G-protein signaling 16; *Cpt1a*, carnitine palmitoyltransferase 1; G-6-P, glucose-6-phosphate; G-3-P, glyceraldehydes-3-phosphate; 1,3-BPG, 1,3-bisphosphoglycerate; 3-PGA, 3-phosphoglycerate; 2-PGA, 2-phosphoglycerate; PEP, phosphoenolpyruvate; Pyr, pyruvate; DHAP, dihydroxyacetone phosphate; Glycerol-3-P, glycerol;-3-phosphate; OAA, oxaloacetate.

In this work we have for the first time provided evidence for possible auto-regulation of *Chrebpβ* through newly identified ChoRE on its proximal promoter and broad consequences of ChREBP on metabolic gene expression (Fig 5). We also described the molecular mechanisms underlying ChREBP-mediated lipid accumulation a phenotype that occurs commonly in pancreatic beta-cells glucotoxicity. We further demonstrated that *Rgs16* is a ChREBP target gene in β-cells with functional *Rgs16* ChoRE sequence, whose activation contributes in part to the lipid accumulation in glucotoxicity.

## Supporting Information

S1 Fig Effect of *Chrebpβ* knockdown on *Rgs16* expression in 832/13 cells. (A) Schematic diagram of tetracycline-inducible lentiviral vectors for expression of microRNA-adapted short hairpin RNA to target *Chrebpβ* (*mirGE-β*) or non-silencing (*mirGE-N*) sequences. (B) Effect of *Chrebpβ* shRNA on the expression of *Chrebpβ* and *Rgs16* in 832/13 cells. We pre-incubated *mirGE-β* cells and *mirGE-N* cells for 24h in RPMI with 5.5 mmol/l D-glucose in the presence of doxycycline 1 μg/mL, and switched to RPMI with 25 mmol/l D-glucose in the presence of doxycycline 1 μg/mL for 48h. The histograms are the means of relative RNA levels normalized to *Ywhaz* and *Hprt1* and expressed as fold activation over the activity seen in *mirGE-N* cells. *, p< 0.05.
(TIF)

S2 Fig The presence of *ChREBPβ* ChoRE sequences on the ChIP-seq peaks. We explored the anti-ChREBP ChIP-seq data using the Integrated Genome Browser and demonstrated the presence of ChoRE sequence identified in this study at the summit of ChIP-seq peaks in mouse liver and white adipose tissue. Gray vertical line indicates the position where previously identified ChoRE is located.
(TIF)
