# Supplementary figures and images for: ChREBP Regulates Itself and Metabolic Genes Implicated in Lipid Accumulation in β–Cell Line
Source: PLoS One. 2016 Jan 25;11(1):e0147411. doi: 10.1371/journal.pone.0147411 (PMC4725739; doi:10.1371/journal.pone.0147411)

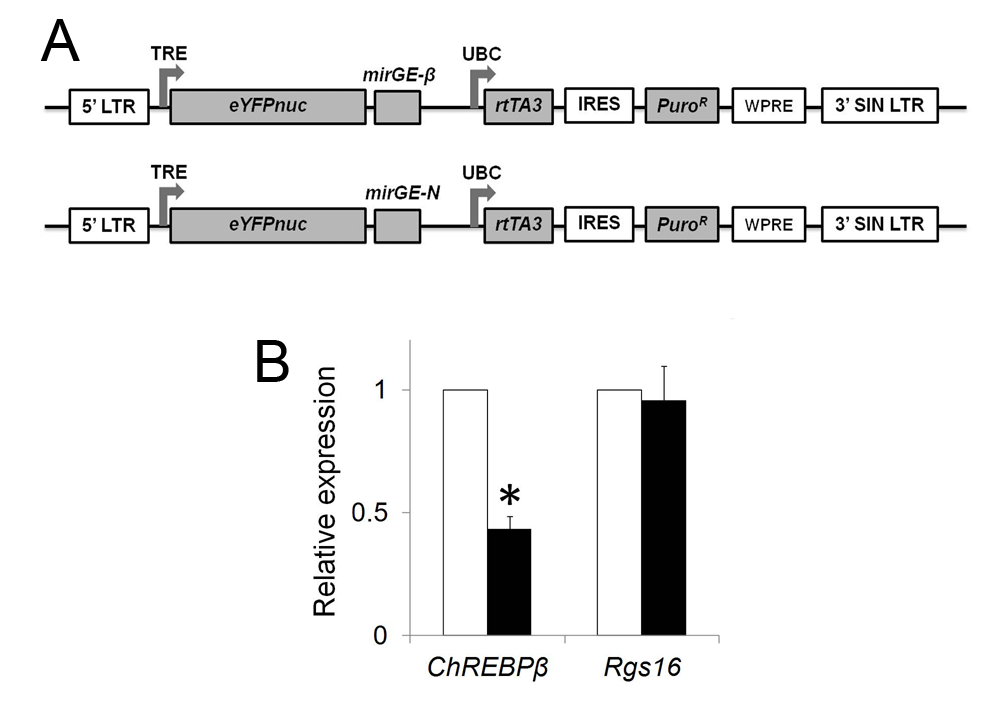

Supplement: S1 Fig — (A) Schematic diagram of tetracycline-inducible lentiviral vectors for expression of microRNA-adapted short hairpin RNA to target Chrebpβ (mirGE-β) or non-silencing (mirGE-N) sequences. (B) Effect of Chrebpβ shRNA on the expression of Chrebpβ and Rgs16 in 832/13 cells. We pre-incubated mirGE-β cells and mirGE-N cells for 24h in RPMI with 5.5 mmol/l D-glucose in the presence of doxycycline 1 μg/mL, and switched to RPMI with 25 mmol/l D-glucose in the presence of doxycycline 1 μg/mL for 48h. The histograms are the means of relative RNA levels normalized to Ywhaz and Hprt1 and expressed as fold activation over the activity seen in mirGE-N cells. *, p< 0.05. (TIF) [file pone.0147411.s001.tif]

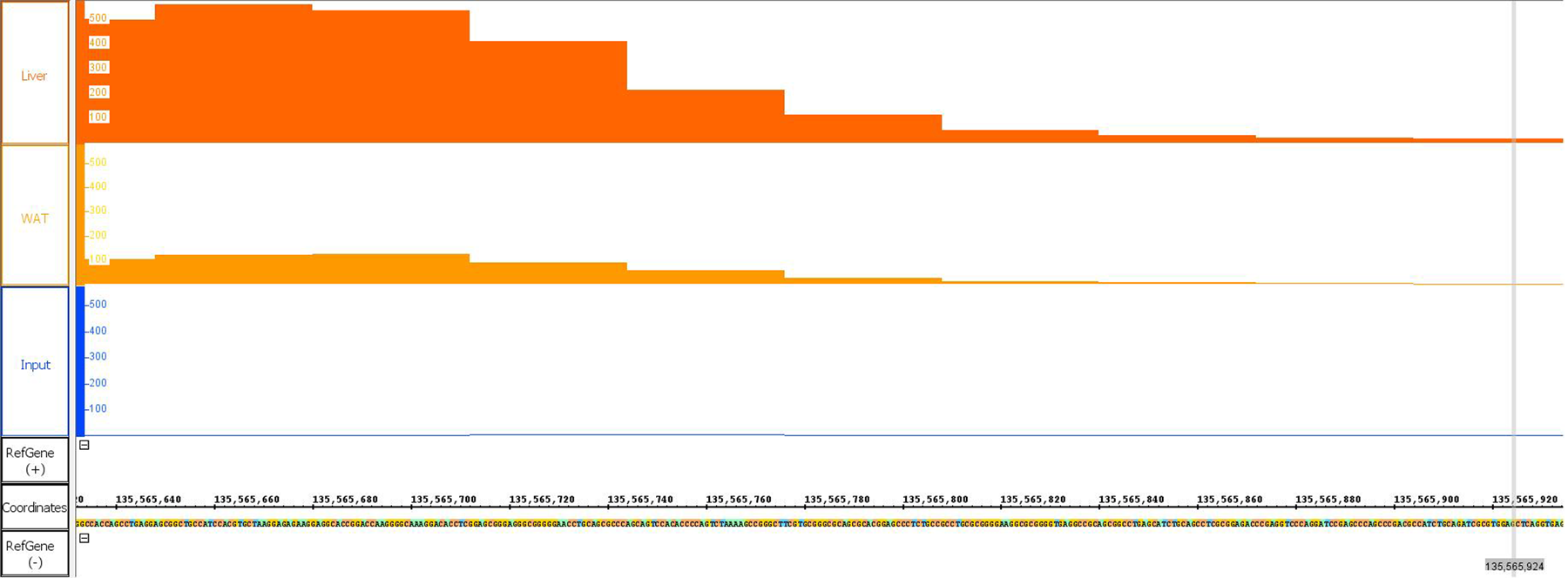

Supplement: S2 Fig — We explored the anti-ChREBP ChIP-seq data using the Integrated Genome Browser and demonstrated the presence of ChoRE sequence identified in this study at the summit of ChIP-seq peaks in mouse liver and white adipose tissue. Gray vertical line indicates the position where previously identified ChoRE is located. (TIF) [file pone.0147411.s002.tif]
